# Gene function annotations for the maize NAM founder lines

**DOI:** 10.1186/s13104-023-06668-6

**Published:** 2024-01-02

**Authors:** Leila Fattel, Colleen F. Yanarella, Blessing Ngara, Olivia T. Johnson, Darwin A. Campbell, Kokulapalan Wimalanathan, Carolyn J. Lawrence-Dill

**Affiliations:** 1 Interdepartmental Genetics and Genomics, Iowa State University, Ames, IA USA; 2 Department of Agronomy, Iowa State University, Ames, IA USA; 3 College of Agriculture and Life Sciences, Iowa State University, Ames, IA USA; 4 Interdepartmental Bioinformatics and Computational Biology, Iowa State University, Ames, IA USA; 5 Department of Computer Science, Iowa State University, Ames, IA USA; 6 College of Liberal Arts and Sciences, Iowa State University, Ames, IA USA; 7 Department of Genetics, Development and Cell Biology, Iowa State University, Ames, IA USA; 8 Neuroscience Team, Atalanta Therapeutics, Boston, MA USA

**Keywords:** Maize, GOMAP, Genomics, NAM, GO, Gene function, Annotation

## Abstract

**Objectives:**

We annotated the latest published sequences of the 26 *Zea mays* Nested Association Mapping (NAM) founder lines using GOMAP, the Gene Ontology Meta Annotator for Plants. The maize NAM panel enables researchers to understand and identify the genetic basis of complex traits. Annotations of predicted functions for genes can help researchers investigate gene-phenotype associations, prioritize candidate genes for phenotypes of interest, and formulate testable hypotheses about gene function/phenotype associations. The creation and release of high-confidence, high-coverage gene function annotation sets for the NAM founder lines is critical to accelerate the generation of knowledge in maize genetics research. GOMAP is a high-throughput computational pipeline that annotates gene functions genome-wide in plant genomes using Gene Ontology functional class terms. Here we report and share GOMAP-generated functional annotations for the NAM founder lines.

**Data description:**

Datasets include the protein sequences used as input, GOMAP-generated annotation files, scripts used to update obsolete terms, and GAF-formatted tab-delimited text files of gene function annotations along with README files that describe formatting, content, and how files relate to each other.

## Objective

GOMAP is an annotation tool that generates high-coverage, high-quality (based on F-measure), whole-genome functional annotations for plants. It assigns genes with Gene Ontology (GO) terms through sequence similarity, domain presence, and mixed method pipelines [1]. The GO framework includes a standardized vocabulary designed to describe gene functions under three categories: biological process, molecular function, and cellular component [2]. Using GOMAP, we annotated the 26 *Zea mays* ssp. *mays* Nested Association Mapping (NAM) founder lines [3]. The maize NAM population was established to enhance the genetic diversity of maize to determine the genetic structure of complex traits by merging the benefits of quantitative trait locus and association mapping studies and reducing their limitations [4].

The availability of the GOMAP-generated *Zea mays* ssp. *mays* NAM founder lines annotation datasets can be of great use to scientists in the plant community, especially those with research focused on maize. GO-based function predictions can allow researchers to identify novel candidate genes for hypotheses generation and testing of gene functions. Moreover, the annotation datasets can also be used for gene-phenotype association analyses, identification of novel genes in a pathway of interest, and investigation of different functions within subpopulations of maize, to name a few. We expect new gene function findings and experimental validations from our cleaned datasets described here.

## Data description

A standardized functional annotation dataset is available for each of the NAM founder lines (Table 1). Datasets include:

- Protein sequences of the maize lines that were used as input for GOMAP. We have included the original protein sequences, the Python script we used to reformat the original sequences to produce the GOMAP input file, and the GOMAP input file. A README file is provided for further description of the data and includes where the original file was downloaded from, and how to run the python file. Reformatting was required for proper text wrapping, removal of any asterisks in the sequences, and the selection of the longest transcript of each gene.
- The raw output gene annotation file produced by GOMAP. This file is the aggregated functional annotation generated by the pipeline and follows the GO Annotation File 2 (GAF 2) format.
- Python scripts and supplementary resources to modify and clean the GOMAP-output file. Modifications are done to the gene and transcript names by adding a transcript identifier column. Cleanup includes the removal of any obsolete GO terms and the removal of duplicates. Descriptions of these files and details on how to run the scripts are provided in an accompanying README file. For consistency, the go.obo file used on all our maize datasets reported here is of release 2022-07-01, the same as that incorporated in GOMAP v1.3.9.
- The final cleaned functional annotation dataset. This is the GAF 2 file that is generated using the 2.3_cleanup.py script. These GO-based gene function predictions can be readily used by the public.

**Table 1:** Overview of data files/data sets

Label	Name of data file/data set^a^	File types (file extension)	Data repository and identifier (DOI or accession number)
B73	Carolyn_Lawrence_Dill_GOMAP_Maize_MaizeGDB_**B73**_NAM_5.0_October_2022_v2.r1	Directory	CyVerse [10] (http://doi.org/10.25739/cfvb-jn16)
B97	Carolyn_Lawrence_Dill_GOMAP_Maize_MaizeGDB_**B97**_NAM_1.0_October_2022.r1	Directory	CyVerse [11] (http://doi.org/10.25739/abf6-pa81)
CML52	Carolyn_Lawrence_Dill_GOMAP_Maize_MaizeGDB_**CML52**_NAM_1.0_November_2022.r1	Directory	CyVerse [12] (http://doi.org/10.25739/qgb3-8743)
CML69	Carolyn_Lawrence_Dill_GOMAP_Maize_MaizeGDB_**CML69**_NAM_1.0_November_2022.r1	Directory	CyVerse [13] (http://doi.org/10.25739/xvga-0f52)
CML103	Carolyn_Lawrence_Dill_GOMAP_Maize_MaizeGDB_**CML103**_NAM_1.0_October_2022.r1	Directory	CyVerse [14] (http://doi.org/10.25739/1n89-rd43)
CML228	Carolyn_Lawrence_Dill_GOMAP_Maize_MaizeGDB_**CML228**_NAM_1.0_October_2022.r1	Directory	CyVerse [15] (http://doi.org/10.25739/e6hc-0406)
CML247	Carolyn_Lawrence_Dill_GOMAP_Maize_MaizeGDB_**CML247**_NAM_1.0_October_2022.r1	Directory	CyVerse [16] (http://doi.org/10.25739/jnwv-g571)
CML277	Carolyn_Lawrence_Dill_GOMAP_Maize_MaizeGDB_**CML277**_NAM_1.0_October_2022.r1	Directory	CyVerse [17] (http://doi.org/10.25739/ggj0-by23)
CML322	Carolyn_Lawrence_Dill_GOMAP_Maize_MaizeGDB_**CML322**_NAM_1.0_November_2022.r1	Directory	CyVerse [18] (http://doi.org/10.25739/36bb-f096)
CML333	Carolyn_Lawrence_Dill_GOMAP_Maize_MaizeGDB_**CML333**_NAM_1.0_November_2022.r1	Directory	CyVerse [19] (http://doi.org/10.25739/tnhe-yr36)
HP301	Carolyn_Lawrence_Dill_GOMAP_Maize_MaizeGDB_**HP301**_NAM_1.0_November_2022.r1	Directory	CyVerse [20] (http://doi.org/10.25739/2jhr-hy41)
Il14H	Carolyn_Lawrence_Dill_GOMAP_Maize_MaizeGDB_**Il14H**_NAM_1.0_November_2022.r1	Directory	CyVerse [21] (http://doi.org/10.25739/t500-af32)
Ki3	Carolyn_Lawrence_Dill_GOMAP_Maize_MaizeGDB_**Ki3**_NAM_1.0_November_2022.r1	Directory	CyVerse [22] (http://doi.org/10.25739/y2t8-zp24)
Ki11	Carolyn_Lawrence_Dill_GOMAP_Maize_MaizeGDB_**Ki11**_NAM_1.0_November_2022.r1	Directory	CyVerse [23] (http://doi.org/10.25739/thx1-dm44)
Ky21	Carolyn_Lawrence_Dill_GOMAP_Maize_MaizeGDB_**Ky21**_NAM_1.0_November_2022.r1	Directory	CyVerse [24] (http://doi.org/10.25739/ay3t-b914)
M37W	Carolyn_Lawrence_Dill_GOMAP_Maize_MaizeGDB_**M37W**_NAM_1.0_November_2022.r1	Directory	CyVerse [25] (http://doi.org/10.25739/cgmt-s267)
M162W	Carolyn_Lawrence_Dill_GOMAP_Maize_MaizeGDB_**M162W**_NAM_1.0_November_2022.r1	Directory	CyVerse [26] (http://doi.org/​​10.25739/pewv-k336)
Mo18W	Carolyn_Lawrence_Dill_GOMAP_Maize_MaizeGDB_**Mo18W**_NAM_1.0_November_2022.r1	Directory	CyVerse [27] (http://doi.org/10.25739/w0zf-jc74)
Ms71	Carolyn_Lawrence_Dill_GOMAP_Maize_MaizeGDB_**Ms71**_NAM_1.0_November_2022.r1	Directory	CyVerse [28] (http://doi.org/10.25739/9gb5-aq74)
NC350	Carolyn_Lawrence_Dill_GOMAP_Maize_MaizeGDB_**NC350**_NAM_1.0_November_2022.r1	Directory	CyVerse [29] (http://doi.org/10.25739/q46m-qy91)
NC358	Carolyn_Lawrence_Dill_GOMAP_Maize_MaizeGDB_**NC358**_NAM_1.0_November_2022.r1	Directory	CyVerse [30] (http://doi.org/10.25739/0w9q-ta36)
Oh7B	Carolyn_Lawrence_Dill_GOMAP_Maize_MaizeGDB_**Oh7B**_NAM_1.0_November_2022.r1	Directory	CyVerse [31] (http://doi.org/10.25739/910q-f303)
Oh43	Carolyn_Lawrence_Dill_GOMAP_Maize_MaizeGDB_**Oh43**_NAM_1.0_November_2022.r1	Directory	CyVerse [32] (http://doi.org/10.25739/8a63-3n35)
P39	Carolyn_Lawrence_Dill_GOMAP_Maize_MaizeGDB_**P39**_NAM_1.0_November_2022.r1	Directory	CyVerse [33] (http://doi.org/10.25739/dgda-md18)
Tx303	Carolyn_Lawrence_Dill_GOMAP_Maize_MaizeGDB_**Tx303**_NAM_1.0_November_2022.r1	Directory	CyVerse [34] (http://doi.org/10.25739/gz5q-rw97)
Tzi8	Carolyn_Lawrence_Dill_GOMAP_Maize_MaizeGDB_**Tzi8**_NAM_1.0_November_2022.r1	Directory	CyVerse [35] (http://doi.org/10.25739/9g8d-ny61)
General file structure within the file for each line	README.txt	.txt	CyVerse
	/0_GOMAP-input	directory	
	0.0_README.txt	.txt	
	0.1_Zm-**line-designation**-REFERENCE-NAM-5.0_**annotation.protein**.fa.gz	.gz	
	0.2_reformat.py	.py	
	0.3_GOMAP-input.fa.gz	.gz	
	/1_GOMAP-output	directory	
	1.0_README.txt	.txt	
	1.1_GOMAP-output.gaf.gz	.gaf.gz	
	/2_cleanup	directory	
	2.0_README.txt	.txt	
	2.1_modify_entity_names.py	.py	
	2.2_GOMAP-output-with-modified-entity-names.gaf.gz	.gaf.gz	
	2.3_cleanup.py	.py	
	/../cleanup_resources	sub-directory	
	go.obo.gz	.obo.gz	
	__init__.py	.py	
	obo_parser.py	.py	
	/3_final-result	directory	
	3.0_README.txt	.txt	
	3.1_Zea_mays_**line-designation**.MaizeGDB.CLEANED.gaf.gz	.gaf.gz	

^a^The terms “line-designation” and “annotation” are a part of nomenclature and are shown in bold font to help the reader see the pattern of the file structure

Each directory has its own README that provides more information about the files. There also is a top-level overall README that describes the dataset more generally. Moreover, each dataset has its own standardized metadata. The datasets are publicly available on CyVerse [5] and can be accessed using the links provided in Table 1.

The structure of our dataset is an attempt to ensure data reproducibility and abidance to the data principles of findability, accessibility, interoperability, and reusability (FAIR) [6]. The overall organization of our annotation datasets is not new; we developed and applied this form for previously studied GOMAP-generated annotation files [7]. A full list of our annotated plant genomes can be found here [8], and includes annotation sets for 24 species, including sorghum, rice, wheat, barley, cotton, and hemp. For users interested in generating their own GO-based annotations, the GOMAP pipeline itself is available for general use [1], and a description of how to use the pipeline is also available [9].

We have generated and publicly released new functional annotations using the most up-to-date version of GOMAP (v1.3.9) on our old datasets, including the previously annotated maize lines Mo17 [36–38], W22 [39, 40], and PH207 [41, 42]. As an example, the annotations for *Zea mays* B73v5 reported here is an update of a previously released dataset [43]. We anticipate that the availability and maintenance of our datasets will benefit researchers in providing plant gene function predictions, paving the way for the generation of testable hypotheses on novel candidate genes of interest.

## Limitations

The quality of the annotations is dependent on the quality of the input sequences. Genomes with high quality sequencing and coverage are expected to have better annotations. However, genomes with lower quality sequencing will result in limitations in downstream analyses.

In the case of the presence of multiple transcripts per gene IDs, GOMAP requires the selection of the longest transcript for each gene ID because the pipeline contains a reciprocal best hit step. This means that not every transcript ID per gene ID is going to be annotated in the resulting file. We have included a transcript ID column in the final cleaned file that allows the user to identify which one was included in the reformatted input file and annotated through GOMAP.

A cleanup step is performed on our GOMAP output file to remove any obsolete GO terms. This step relies on using a go.obo file. For the datasets reported here, we have used the go.obo file released in 2022-07-01. A user may replace this with the most current version of the go.obo file currently available for their own output.

While using the functional annotation datasets, it is worth noting that the GO Directed Acyclic Graph (DAG) lacks a good portrayal of plant functions underrepresented in the model species, *Arabidopsis thaliana*. This could lead to instances where the assignment of unconventional functions is due to the absence of related plant functions [7].

## Data Availability

The data described in this Data note can be freely and openly accessed on CyVerse under the DOIs listed in Table 1. Please see Table 1 and the reference list for details and links to the data.

## Abbreviations

GOMAP: Gene Ontology Meta Annotator for Plants
NAM: Nested Association Mapping
GO: Gene Ontology
DAG: Directed Acyclic Graph
FAIR: Findability, accessibility, interoperability, and reusability
